# Dedifferentiated liposarcoma found in ovary: A rare case report

**DOI:** 10.1097/MD.0000000000030691

**Published:** 2022-09-16

**Authors:** Junyu Chen, Lianying Ding, Taiwei Wang, Mengqi Wang, Shuhua Zhao, Yang Xia

**Affiliations:** a Departments of Gynecology and Obstetrics, The Second Hospital of Jilin University, Changchun, China; b Central Hospital of Zibo, Zibo, China; c Department of Rehabilitation, Nursing School of Jilin University, Changchun, China; d Department of Pathology, The Second Hospital of Jilin University, Changchun, China.

**Keywords:** dedifferentiated liposarcoma, mesentery, ovary

## Abstract

Dedifferentiated liposarcoma (DL) of the ovary is a rare mesenchymal tumor which is difficult to diagnose before surgery. The diagnose of DL was depended on the morphology, Immunohistochemical stains, and fluorescence in situ hybridization after the complete surgical resection.

**Patient concerns:**

We report a case of 63-year-old postmenopausal female who presented with a large pelvic mass and was initially thought to be an original ovarian tumor. However, it was subsequently considered to be a DL arising from the mesentery and developing in ovary.

**Diagnosis:**

Ovarian DL.

**Interventions:**

Bilateral salpingo-oophorectomy.

**Outcomes:**

Until now, there is no recurrence symptoms represented in the patient after surgery for 12 months.

**Lessons:**

DL happening in ovary is rare. In this case, the exact origin of the mass still remains ambiguous because of the lack of morphological evidence. Although retroperitoneum is the most common area of DL origin, we for long suspect that the mass was initially derived from mesentery and developed in ovary. A long-term may help confirm the exact origin of the tumor. Complete surgical resection plays an important role in the treatment.

## 1. Introduction

Soft tissue sarcomas are a class of rare malignant tumors originating from mesenchymal tissues.^[1]^ Liposarcoma, arising from adipose cells, is the most common soft tissue sarcomas, with approximately 52% occurring in the extremities, 42% developing in the retroperitoneum (RP), and 6% deriving from the head and neck.^[2]^ The histological classification of liposarcoma could be divided into five groups: well-differentiated liposarcoma (including adipocytic, sclerosing, inflammatory, and spindle-cell), marked by ring or long chromosomes; myxoid liposarcoma; round cell liposarcoma, characterized by reciprocal translocation t(12;16)(q13;p11); pleomorphic liposarcoma, characterized by complex karyotypes; and dedifferentiated liposarcoma (DL).^[3–5]^

DL, a variant type of liposarcoma, was featured as a combination of low-grade nonlipogenic morphology, high-grade nonlipogenic morphology, and well-differentiated liposarcoma. Moreover, the transition from low-grade to high-grade nonlipogenic morphology could also be observed.^[1,3]^ Commonly, DL was reported to occur in the RP, while an ovary location is extremely rare. The clinical outcome of DL remains controversial. A previous study found that DL is less aggressive than that in other high-grade pleomorphic sarcomas.^[3]^ However, DL was considered as a disease with aggressive behavior and poor prognosis in recent studies.^[6]^ The prognostic factors of DL are also related to tumor size, anatomical location, and surgical resection.^[6,7]^

We reported herein a case diagnosed as DL that occurred in ovary showing an amplification of the murine double minute 2 (MDM2) gene as well as the high expression of other DL related proteins.

We present the following case in accordance with the CARE reporting checklist. The study was approved by the Ethics Committee of the Second Hospital of Jilin University (approval no. 2019084). Written informed consent was obtained from the patient.

## 2. Case presentation

A 63-year-old postmenopausal female visited the department of Obstetrics and Gynecology of Jilin University Hospital with complaints of a palpable pelvic mass found by herself 19 days prior. The patient was married at the age of 23 and had a history of gravida 2 and para 1. She suffered from a resection of brain tumor in 2000, a hysterectomy in 2011, and a resection of brain tumor because of recurrence in 2019. On gynecological and pelvic examination, an approximately 20 gestational week-sized mass, with restriction mobility, irregular form, and unclear boundary, was palpable in the meso-pelvic cavity. There were no laboratory abnormalities. The tumor markers such as Pro-gastrin-releasing peptide, alpha fetoprotein, cancer antigen 125, cancer antigen 199, and human epididymal protein 4 were within their normal ranges.

**Figure 1.**
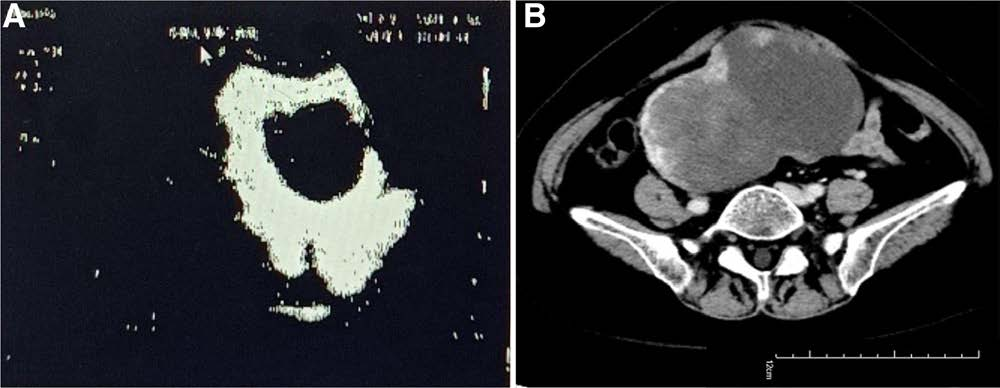
Abdominal ultrasonography (A) and computed tomography (B) presenting a large mass in the pelvis.

The mass was suspected as an ovary-derived tumor based upon pelvic contrast-enhanced computed tomography (CT) and abdominal ultrasonography. The ultrasound revealed a hypoechoic and irregular solid tumor with blood flow signals in the edge, measured approximately 17.0 × 9.3 cm, occupying the middle of the pelvic cavity (Fig. 1A). The enhanced CT scan (Fig. 1B) showed that the large mass was 13.2 × 8.6 cm in size with heterogeneous density (the value of CT was about 33 HU) and part of lobulated mass could be observed. Enhancement of the edge of the mass was significant, but there was no obvious enhancement in the internal component of the mass. Multiple lymph node smaller than 6 mm were observed with a slight enhancement.

During the laparotomy, a large, firm mass with irregular shape was found in the middle of pelvic cavity. The mass, featured with thick blood vessels on the smooth surface with a complete capsule, was seemed to adhere densely to the intestinal tube and mesentery. While continuing the exploration along the root of the tumor, we found that the right ovary was encapsulated by the mass, and the infundibulopelvic ligament was the common root of the mass and right ovary. It seemed that the mass was originated from right ovary. In consideration of the age of the patient and preventing the recurrence of the tumor, bilateral salpingo-oophorectomy was performed. There were no other suspicious malignant lesions in the pelvic cavity.

**Figure 2.**
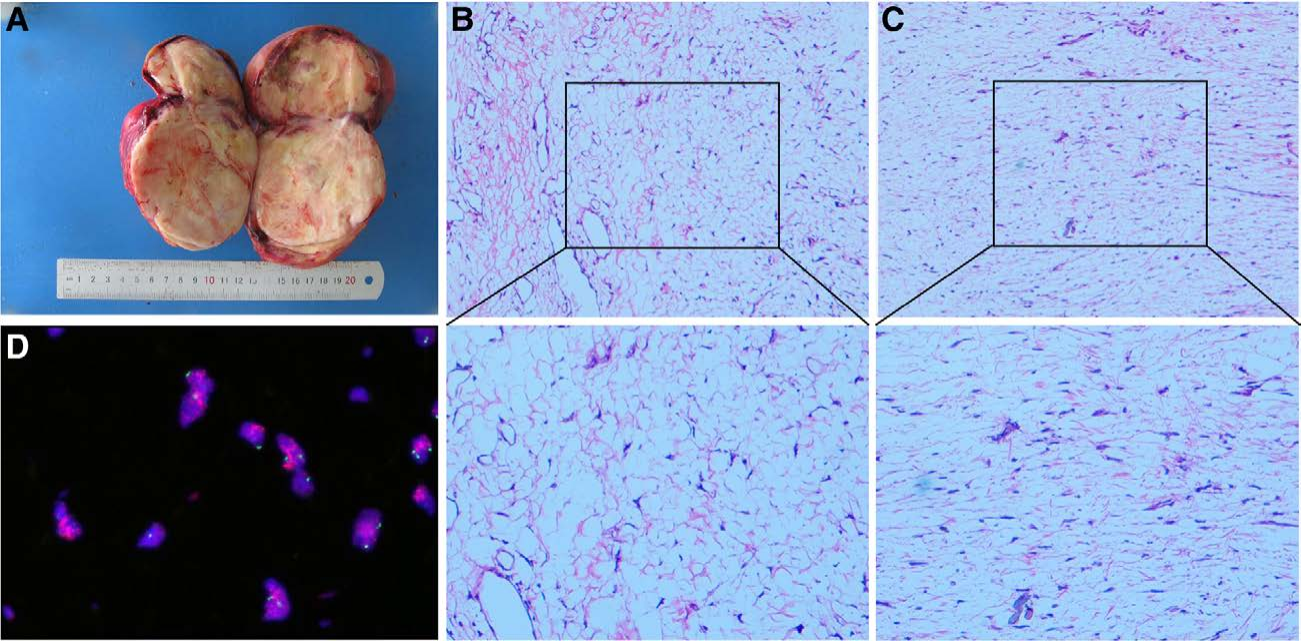
(A) The opened specimen of dedifferentiated liposarcoma showing the inner morphology of the mass. (B and C) H&E staining showing the histopathology of the tumor cells (×100, ×200). The slide was observed under an inverted optical microscope (Motic, AE2000). (D) The fluorescence in situ hybridization slide was examined under Nikon fluorescence microscope (Ni-U, ×100, green for CEP17, red for murine double minute 2, and blue for nucleus). CEP17 = chromosome enumeration probe for Chromosome 17.

Macroscopic examination of the specimen presented an irregular and solid mass with a whitish and yellowish cut surface and measuring approximately 16 × 13 × 9 cm (Fig. 2A). Microscopic examination (H&E staining) showed that the morphology of the tumor was low-medium grade, and the cells were spindle and fibrosarcomatoid. No obvious fatty tumor component was observed (Fig. 2B and C).

**Figure 3.**
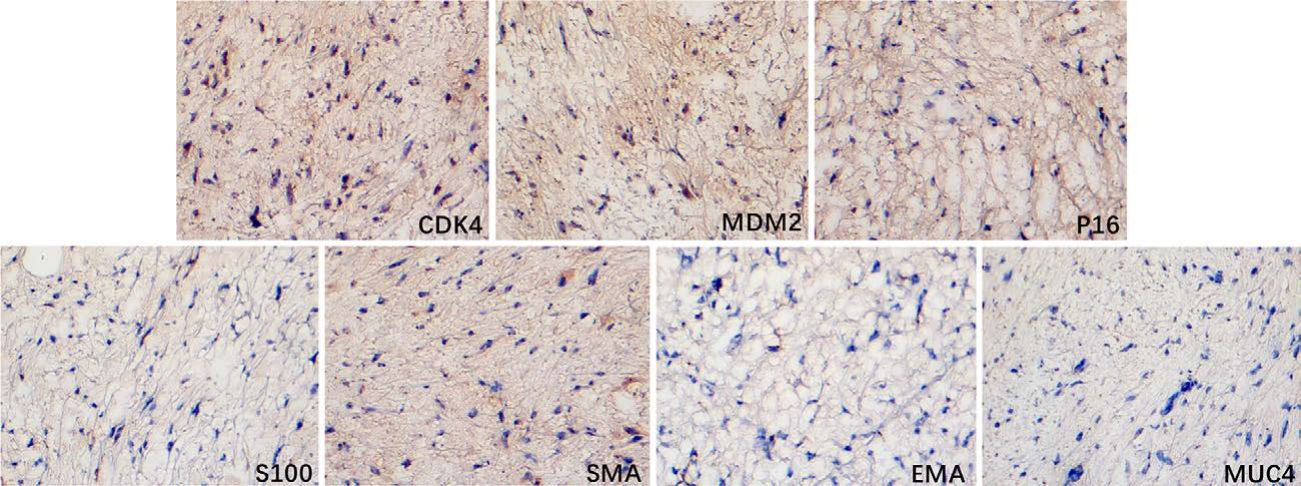
Immunohistochemical staining for CDK4, murine double minute 2, P16, S100, SMA, EMA, and MUC4 in the dedifferentiated liposarcoma tissue (×200). The slide was observed under an inverted optical microscope (Motic, AE2000). Brown staining indicated the positive cells. CDK4 = cyclin dependent kinase 4, EMA = epithelial membrane antigen, MUC4 = mucin 4, P16 = cyclin dependent kinase inhibitor 2A, S100 = S100 calcium binding protein B, SMA = smooth muscular actin.

Immunohistochemical (IHC) stains for CK (AE1/AE3), ER, PR, Calretinin, epithelial membrane antigen, S100 calcium binding protein B, ALK, α-inhibin, and mucin 4 were negative, while MDM2, cyclin dependent kinase 4 (CDK4), cyclin dependent kinase inhibitor 2A (P16), INI-1, BRG-1, WT1, and CD10 were positive. Some other related molecules, such as smooth muscular actin, DES, Ki67, CD34, CD99, PBH3, and CyclinD1, showed weakly positive. Part of representative pathology images were showed in Figure 3. Furthermore, fluorescence in situ hybridization (FISH) analysis revealed an amplification of the MDM2 gene in the mass (Fig. 2D; Table 1).

**Table 1:** FISH detection report of MDM2 gene amplification.

Probe	Analysis of cell number	MDM2 gene copy number	CEP17 copy number	MDM2/CEP17	MDM2 gene amplification result
MDM2 dual color separation fluorescent probe	100.0	25 (cluster)	2.0	12.50	Positive

FISH = fluorescence in situ hybridization, MDM2 = murine double minute 2.

**Figure 4.**
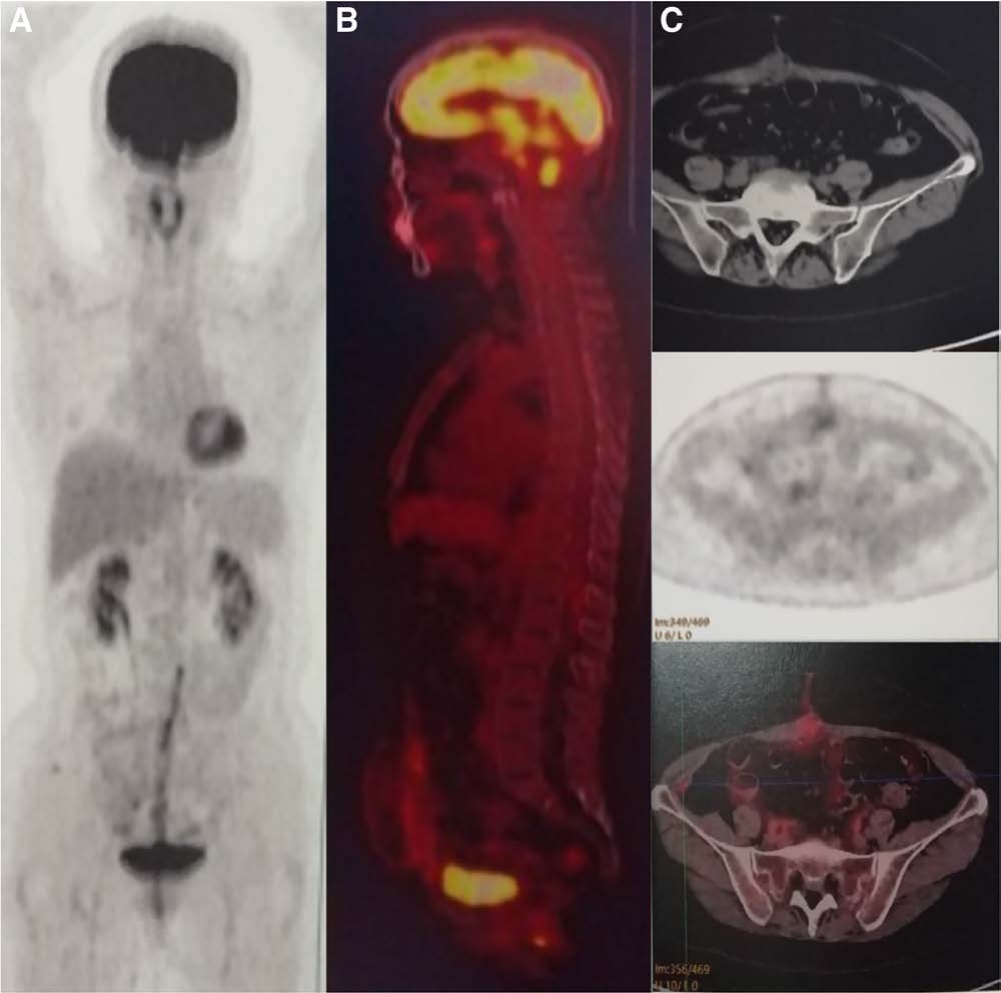
PET-CT showed that there were no abnormal FDG and swollen lymph node in the retroperitoneal and no metastasis lesions on the other organs. (A) The coronal image of PET-CT for the whole body. (B) The sagittal image of PET-CT for the whole body. (C) The cross-sectional image of PET-CT for the abdomen. FDG = fuorodeoxyglucose, PET-CT = positron emission tomography-computed tomography.

After 15 days of surgery, positron emission tomography-CT (PET)-CT was performed to evaluate whether there are metastasis lesions or remnant tissues. The report of PET-CT demonstrated that fuorodeoxyglucose intake was slightly increased in pelvic medial abdominal wall, left pelvic peritoneum, and median abdominal wall, which was considered as postoperative inflammation. There were no metastasis lesions on the other organs. Moreover, there was no abnormal fuorodeoxyglucose and swollen lymph node in the retroperitoneal (Fig. 4).

## 3. Discussion

DL is a subtype of liposarcoma, which was rarely reported to develop in ovary. In this case, because there were no characteristic symptoms and laboratory results, it is difficult for us to confirm the properties of the mass. Although preoperative ultrasound and CT revealed that the mass was located in the pelvic, its exact position and origin remained unclear. Accurate diagnosis still demands on the operation and pathology.

During the surgery, we thought that the mass had a close relationship with the right ovary as well as the mesentery. To previous reports, most of DLs are derived from RP and pelvic soft tissue, followed by limbs, spermatic cord, head, and neck.^[8]^ A few cases reported that DL could be found in peritoneum, particularly in mesentery or sigmoid mesocolon.^[1]^ One case has reported an ovary-derived DL which, however, was subsequently suspected to be a retroperitoneal DL.^[2]^ In this case, the exact origin of the mass still remains ambiguous because of the lack of morphological evidence. Although RP is the most common area of DL origin, we still highly suspected that the mass was initially derived from mesentery and developed in ovary. The reasons for this suggestion are as follows: during the laparotomy, the mass seemed to adhere densely to the intestinal tube and mesentery although its smooth capsule was complete; after separation of adhesion, we found that the mass and right ovary have the same root which connected to the infundibulopelvic ligament; postoperative PET-CT did not find any suspicious metastasis or remnant lesions in the RP. However, a long-term follow-up may help confirm the exact origin of the tumor. Because there is no treatment guideline for differentiated liposarcoma happening in ovary, we just prudently referred the patient for chemotherapy and radiotherapy after the surgery. However, the patient refused our suggestion and just chosen to follow-up timely. Until now, there is no recurrence symptoms represented in the patient after surgery for 12 months.

Actually, this case should be distinguished with well-differentiated liposarcoma with myxoid changes and myxoid liposarcoma. The basics of differential diagnosis are as follows: The differential diagnosis from well-differentiated liposarcoma with myxoid changes. After being carefully observed, neither typical well-differentiated liposarcoma ingredients nor at least piece distribution of adipose tissue and adipoblast were found in any slices. Histologically, it showed basically the same spindle cell sarcoma morphology, and mucilaginous degeneration of the stroma is considered as a nonspecific change. These characteristics did not accord with the diagnosis of well-differentiated liposarcoma; the differential diagnosis from myxoid liposarcoma. In this case, although the stroma was myxoid, there was neither adipoblastic component nor characteristic plexiform or branched vessels, which did not support the diagnosis of myxoid liposarcoma. The amplification of MDM2 gene identified by FISH in this case was not a specific genetic change of myxoid liposarcoma.

Microscopically, DL was commonly formed with two major components: highly differentiated liposarcoma and dedifferentiated components. However, there is no obvious transition process and the boundary is clear.^[8,9]^ Immunohistochemistry and FISH are two important tools in the diagnosis of DL. The combined detection of CDK4, MDM2 and P16 plays an important role in the definite diagnosis.^[10]^ Identifying MDM2 amplification by FISH was considered as a golden standard for diagnosis of DL. Importantly, this case presented spindle cell sarcoma with a few pleomorphic cellular components. Although no typical well-differentiated liposarcoma component was observed, MDM2, CDK4 and P16 IHC staining were diffusely positive, and MDM2 gene amplification, identified by FISH, supported the diagnosis of DL. Considering that in some cases of DL featured as completely sarcomatous, where only few or no well-differentiated liposarcoma components could be found, the absence of well-differentiated liposarcoma components cannot rule out the diagnosis of DL. Moreover, this case has no characteristic histological morphology. After IHC staining, the possible sources of epithelial origin, smooth muscle and striated muscle origin, peripheral nerve origin, and ovarian sex cord mesenchymal origin were excluded, The other tumors were basically excluded by the results of IHC staining.

Due to the low incidence of ovarian DL, its exact origin and pathogenesis are still unclear and needed more clinical case report to further discuss. However, this case indicated that complete surgical resection still played an important role in the strategy of treatment, and could be applied in the similar patients in the future. Furthermore, the necessity of chemotherapy and radiotherapy in the disease needs more clinical cases to determine.

## Author contributions

JC, TW, MW, and SZ wrote the main manuscript text. LD and YX prepared Figures 1–4 and Table 1. All authors reviewed the manuscript.

**Conceptualization:** Junyu Chen, Lianying Ding, Taiwei Wang, Mengqi Wang, Shuhua Zhao, Yang Xia.

**Data curation:** Junyu Chen, Lianying Ding, Taiwei Wang.

**Formal analysis:** Junyu Chen, Lianying Ding.

**Methodology:** Junyu Chen.

**Supervision:** Shuhua Zhao, Yang Xia.

**Writing – original draft:** Junyu Chen, Lianying Ding, Taiwei Wang, Mengqi Wang, Shuhua Zhao.

**Writing – review & editing:** Taiwei Wang, Mengqi Wang, Shuhua Zhao, Yang Xia.
